# Limbic gray matter increases in response to cognitive-behavioral therapy in major depressive disorder

**DOI:** 10.1038/s41398-025-03545-7

**Published:** 2025-08-27

**Authors:** Esther Zwiky, Tiana Borgers, Melissa Klug, Philine König, Konrad Schöniger, Janine Selle, Antonia Küttner, Luisa Brunner, Elisabeth J. Leehr, Udo Dannlowski, Verena Enneking, Ronny Redlich

**Affiliations:** 1 Department of Psychology, University of Halle, Halle, Germany; 2 German Center for Mental Health (DZPG), Halle-Jena-Magdeburg, Halle, Germany; 3 Institute for Translational Psychiatry, University of Münster, Münster, Germany; 4 Center for Intervention and Research on adaptive and maladaptive brain circuits underlying mental health (C-I-R-C), Halle-Jena-Magdeburg, Halle, Germany

**Keywords:** Human behaviour, Neuroscience, Depression

## Abstract

Major depressive disorder (MDD) is related to volumetric decreases in (cortico-)limbic brain regions. In contrast to pharmacological and electroconvulsive therapy, little is known about the brain structural effects of psychotherapy and potential links to symptom improvements. In a naturalistic longitudinal study using structural magnetic resonance tomography, gray matter volume (GMV) and clinical measures were assessed in 30 outpatients with MDD before and after 20 cognitive-behavioral therapy (CBT) sessions. Data from 30 healthy controls was acquired. Region-of-interest analyses revealed significant GMV increases within patients for the right anterior hippocampus and the bilateral amygdala, resulting in a significant group-by-time interaction for the left amygdala (*p* ≤ 0.022). Simultaneously, analyses revealed volumetric decreases in the right posterior hippocampus (*p* = 0.016). While there were no associations with overall symptom improvement, right amygdala volume increases were slightly associated with improvements in identifying feelings (*r*_s_ = 0.321, *p* = 0.042). Together, findings show an impact of CBT not only on psychopathology but also on brain structure. The connection between CBT-related increased amygdala GMV and improved emotion identification emphasizes the role of improvements in emotional awareness.

## Introduction

Major depressive disorder (MDD) is a significant public health concern and one of the most prevalent and disabling mental disorders. Globally, around 280 million people are suffering from MDD, which is the second leading cause of years lived with disability [1]. Besides alterations on a behavioral and brain functional level [2], MDD is also associated with brain structural changes [3–6]. Several researchers have reported structural alterations in brain areas connected to emotion processing [3, 4, 7, 8]. Especially in (cortico-)limbic regions (e.g., orbitofrontal cortex (OFC), cingulate cortex, hippocampus, amygdala), except for some evidence for enlargement of amygdala volume (e.g., [9–11]), most previous studies have found volumetric decreases in comparison to healthy individuals [4, 12, 13].

Although psychotherapy is recommended as a stand-alone or combined treatment for MDD [14], in contrast to somatic treatments, such as electroconvulsive therapy (ECT) and antidepressant medication [15], little is known about its longitudinal effects on gray matter volume (GMV). According to Eric Kandel’s fifth principle of his framework for psychiatry and neuronal sciences, every psychotherapy capable of inducing long-lasting behavioral changes does so by modifying the brain structure [16]. However, the evidence for the neuroplasticity effects of psychotherapy in depressive disorders is strongly limited. Du et al. [17] found local GMV increases in the right middle frontal gyrus (MFG) and decreases in the left postcentral gyrus in subclinical depressive college students after a 4-week group CBT program. Another study by Meng et al. [18] reported normalization of the nucleus accumbens volume in patients with mild to moderate depression after eight weeks of a manualized CBT intervention. Since these studies have focused on short-term interventions in a group setting for a subclinical population [17] or lack of involvement of psychotherapists [18] generalizability for structural plastic effects of psychotherapy in general or CBT in MDD is questionable, and more ecologically valid studies are required.

If treatments can have a brain structural impact in patients, it is of particular interest whether these changes are related to symptom improvement. Brain structural changes achieved by antidepressants were correlated with depressive symptom improvement [19], while they tended to be unrelated to ECT response in several studies [15, 20]. For their CBT group program, Du et al. [17] demonstrated a relationship between an increase in MFG volume and a subjective improvement in overall depressive symptoms. However, MDD is linked to a variety of psychological dysfunctions, resulting in a substantial variance in possible symptom profiles [21], which are connected to distinct brain structural abnormalities [22]. In other words, there is no single “depression area” in the brain that can eliminate all depressive symptoms if precisely targeted. Given this heterogeneity in clinical and corresponding neurobiological phenotypes, it seems likely that regional volumetric changes are more closely linked to changes in particular (dys)functions rather than to improvements in overall MDD symptoms.

Since brain structural alterations in MDD are primarily observed in (cortico-)limbic brain regions, which are heavily involved in processing and regulating emotions, emotional dysfunctions could be of interest. In particular, alexithymia - a psychological concept referring to difficulty in recognizing and expressing emotions - may play a noteworthy role, also considering its well-documented links to depression and its brain structural correlates. Alexithymia is defined as a lack of ability to identify and describe one’s emotions (affective component) and having a more externally oriented cognitive style (cognitive component; [23]). A robust moderate correlation between the self-reported overall and affective components of alexithymia and depression has been reported in the literature [24]. In an investigation on sub-clinical depression, CBT-dependent improvements in alexithymia were linked to declines in depressive symptoms [25]. According to Preece et al. [26], the affective alexithymic components manifest in the misappraisal of emotions, leading to maladaptive emotion regulation and ultimately causing depressive symptoms. Indeed, alexithymia has been linked to several maladaptive features and behaviors in depressive samples, like suicidal ideation [27], self-harm [28], problematic internet use [29], and melancholic features and somatization [30], for example. Additionally, there is an overlap in the brain structural foundation of alexithymia and depression, as alexithymia is also linked to volumetric decreases in (cortico-)limbic brain regions that play a role in emotional experience (e.g., insula, amygdala, OFC; [31]). Even though alexithymia seems highly relevant in the psychopathology and neurobiology of MDD, it is often overlooked since, unlike other dysfunctions (e.g., anhedonia, executive dysfunctions), it is not acknowledged by the MDD diagnosis criteria and instruments.

Generally, the present study aims to investigate longitudinal volumetric brain alterations after 20 CBT sessions and their potential relationship to clinical outcomes in a sample of 30 patients with MDD. Only limited evidence of brain structural effects of CBT in MDD exists so far, and previous studies lack generalizability. We choose a naturalistic approach by recruiting outpatients who attend a CBT carried out by licensed psychotherapists or supervised psychotherapists in training, and by the guidelines of the German public healthcare system to enhance ecological validity. Regional levels of GMV are compared to a group of 30 healthy controls by following a mixed-design analysis of variance model. We aim to concentrate on brain structural effects in (cortico-)limbic brain regions by applying a regions-of-interest (ROI)-based approach. Although volumetric alterations in MDD have been mainly observed in (cortico-)limbic areas, there are no investigations on the impact of psychotherapy (or CBT) on GMV in these areas. Secondly, we aim to investigate potential links between clinical and brain structural effects of CBT. Besides overall symptom severity, we also focus on dysfunctions in alexithymia, given its previously reported shared regional pattern of GMV decreases with depression and its predictive quality for depressive symptoms and treatment outcomes. We expect volumetric increases through CBT in (cortico-)limbic brain areas in the patient group (hypothesis 1). We further hypothesize regional volumetric changes to be related to improvements in alexithymia rather than declines in overall depressive symptom severity (hypothesis 2).

## Materials and methods

### Participants and study design

The sample of the present longitudinal study included 30 MDD patients treated with CBT and 30 healthy controls (HC). All measures were acquired at two time points: before the beginning of the CBT (t1) and after 20 CBT sessions (t2) or in an equivalent time interval for HC (time interval between t1 and t2 in weeks: *M*_patients_ = 39.57 (*SD* = 18.64), *M*_HC_ = 34.07 (*SD* = 6.98)). Patients were recruited through the psychotherapeutic outpatient unit of the University of Muenster. At t1, all patients were either on the waiting list or in the trial phase for CBT, with a mean time interval between t1 and treatment start of 19.19 days (*SD* = 16.79 days). All patients received CBT for depressive disorders, carried out by licensed psychotherapists or supervised psychotherapists in training. Due to the naturalistic study design, CBT followed a natural course but always met the standards of the national care guidelines for unipolar depression. As it was funded by the German public healthcare system, CBT was carried out following its legal guidelines. HC were recruited through public notices and newspaper announcements. At both time points, participants completed a structural MRI session, a clinical interview for DSM-IV (SKID-I; [32]), the Hamilton Depression Rating Scale (HDRS; [33]), as well as the Beck Depression Inventory (BDI; [34]) for overall depressive symptoms. To assess alexithymia, we administered the Toronto Alexithymia Scale (TAS20; [35]), which differentiates three domains of alexithymia (Difficulty Identifying Feelings (DIF), Difficulty Describing Feelings (DDF), Externally Oriented Thinking (EOT)) and a sum score (TAS20 sum). General exclusion criteria were any neurological abnormalities, organic mental disorders, bipolar or psychotic disorders, substance dependency, brain injuries, or MRI contraindications, and age outside the range of 18–65 years. Patients were included if they were in an acute or partially remitted depressive episode and fulfilled the criteria of MDD according to DSM-IV. HC were free of any lifetime psychiatric diagnosis and current psychotropic medication. A BDI score >9, indicating at least mild to moderate depression according to the Center for Cognitive Therapy guidelines for patients with affective disorders [36], was an inclusion criterion for depressed patients but an exclusion criterion for HC at both time points. Nine patients (30.0%) reported an antidepressant intake. To account for the effects of medication, we computed a medication load index for each patient, following the approach of Hassel [37]. In the patient group, there were slightly more males than females (46.7%), while females (66.7%) predominated in the HC group (*p* = 0.118, *ns*). Both groups had relatively high levels of education (average years of education: *M*_patients_ = 13.47, *M*_HC_ = 14.41, *p* = 0.170, *ns*). However, the average age was significantly higher in the HC group (*M*_patients_ = 28.10 years, *M*_HC_ = 43.23 years, *p* < 0.001, *d* = 1.23). For detailed sample characteristics, see Table 1. All procedures comply with the ethical standards of the relevant national and institutional committees on human experimentation and the Helsinki Declaration of 1975, as revised in 2008. The experimental procedure was approved by the local Institutional Review Board (IRB; Amendment of 2016-173-f-S; 2020-205-f-S). All participants gave written informed consent and received financial compensation.

**Table 1:** Sociodemographic and clinical characteristics at baseline (t1).

	patient sample (*n* = 30)	HC sample (*n* = 30)	*t*-test/χ^2^-test	*p*
	*M* (*SD*)	*M* (*SD*)		
*Sociodemographic characteristics*				
age	28.10 (8.38)	43.23 (15.27)	*t*(58) = 4.76	<0.001
sex (m/f)	16/14	10/20	χ^2^(1) = 2.44	0.118
years of education (*n* = 30/*n* = 29)	13.47 (2.21)	14.41 (2.98)	*t*(57) = 1.39	0.170
*Longitudinal interval*				
time interval (t1 to t2) in weeks	39.57 (18.64)	34.07 (6.98)	*t*(58) = −1.51	0.136
number of CBT sessions at t2	22.50 (3.72)			
*Psychometric Scales*				
BDI	23.37 (10.27)	2.47 (2.92)	*t*(58) = −10.72	<0.001
HDRS	13.53 (5.73)	2.27 (2.93)	*t*(58) = 9.58	<0.001
TAS20				
sum	54.45 (11.12)	-	-	-
DIF	20.27 (6.33)	-	-	-
DDF	15.43 (4.18)	-	-	-
EOT	18.77 (4.06)	-	-	-
*Clinical characteristics*				
medication load index				
0 = absent	21 (70.0%)	-	-	-
1 = low	2 (6.7%)	-	-	-
2 = high	7 (23.3%)	-	-	-
DSM-IV diagnosis				
MDD, single episode	13 (43.3%)	-	-	-
MDD, recurrent	17 (56.7%)	-	-	-
degree of remission				
acute	24 (80.0%)	-	-	-
in partial remission	6 (20.0%)	-	-	-
acute comorbidity (yes/no)	15/15	-	-	-

*N* = 60.

*CBT* cognitive-behavioral therapy, *BDI* Beck’s Depression Inventory, *HDSR* hamilton-depression-rating-scale, *TAS20* Toronto alexithymia scale, *DIF* difficulty identifying feelings, *DDF* difficulty describing feelings, *EOT* externally-oriented thinking.

### Structural MRI: data acquisition and preprocessing

T1-weighted high-resolution anatomical images were acquired using a research-dedicated 3-Tesla-MRI (Prisma, Siemens, Erlangen, Germany) with a 3D magnetization prepared rapid acquisition gradient echo sequence (MPRAGE), TR = 2130 ms, TE = 2.28 ms, TI = 900 ms, FA = 8°, voxel size=1.0×1.0×1.0 mm³, Acquisition Direction Sagittal, 192 slices. Structural images were longitudinally preprocessed using the CAT12-toolbox (version 12.7 [revision1615], http://www.neuro.uni-jena.de/cat12-html/cat.html). Images were bias-corrected, tissue classified, and normalized to MNI-space using linear (12-parameter affine) and non-linear transformations, including high-dimensional normalization using Shooting-Registration. Gray matter (GM) segments were modulated by non-linear components only to preserve actual GM values locally (modulated GMV). The modulated GM images were smoothed with a Gaussian kernel of 8 mm full-width half maximum (FWHM).

### Statistical analysis

#### Sociodemographic and clinical data

Clinical data were analyzed using SPSS Statistics (version 27.0; IBM Corporation). To prove the clinical efficacy of CBT, paired *t*-tests (*p* < 0.05, two-tailed) were performed, which compared the baseline (t1) overall depressive symptom burden (BDI, HDRS) to BDI and HDRS scores after 20 CBT sessions (t2). The procedure was repeated for the four TAS20 scales. Paired *t-*tests were sensitive to detect effects with a medium effect size of *d* ≥ 0.529 (analysis performed in G*Power 3.1.9.7 with α = 0.05, β = 0.80, *n* = 30). In addition, frequencies of changes in the degree of remission of the depressive episode according to DSM-IV criteria were analyzed. Subsequently, we performed non-parametric Spearman correlation analyses (*p* < 0.05, one-tailed) to investigate the association between alexithymia and depressive symptoms at baseline (t1).

#### Statistical analyses of MRI data

MRI data analyses were carried out using statistical parametric mapping software (SPM12, Welcome Department of Cognitive Neurology, London, UK; http://www.fil. ion.ucl.ac.uk/spm). First, in order to detect potential CBT effects on brain structure, a 2×2 mixed ANOVA was applied, with group (patients vs. HC) as a between-subjects factor and time (t1 vs. t2) as a within-subjects factor using a full factorial model in SPM. In order to test hypothesis 1, we performed paired *t*-tests to compare GMV differences in the patient group throughout CBT (t1 vs. t2). Differences in GMV between patients and HC at baseline (t1) and after CBT (t2) were tested via two-sample *t*-tests. According to our objective, ROI analyses of the bilateral OFC, the bilateral amygdala, the bilateral insula, and the bilateral hippocampus were performed. All ROIs were created by means of the Wake Forest University PickAtlas [38] according to the AAL-atlas definitions [39]. To account for multiple testing, significance thresholds for multiple testing were obtained at the cluster level by threshold-free cluster enhancement (TFCE), implemented in the TFCE toolbox (http://dbm.neuro.uni-jena.de/tfce, version 232). A conservative family-wise error (FWE)-corrected threshold of *p* < 0.05 (one-tailed) was established, obtained by 5000 permutations per test (non-parametric). Age, sex, and total intracranial volume (TIV) were included as covariates of no interest. We repeated the procedure at a whole brain level for exploratory reasons.

Second, to address hypothesis 2, GMV values of clusters showing significant GMV changes within patients were extracted for further analyses in SPSS. To investigate associations between regional GMV changes and symptom changes in patients, we computed scores for the change (Δ) in GMV, overall depressive symptom measures, and the TAS-20 scales. For a more straightforward interpretation, change score values greater than 0 always indicate an increase in GMV or an improvement in symptoms. Therefore, we calculated change scores using different formulas based on the polarity of measurements. For GMV, we used the formula Δ=t2-t1, while for BDI, HDRS, and TAS20, we used the formula Δ=t1-t2. Subsequently, we calculated non-parametric Spearman correlations (*p* < 0.05, one-tailed, unadjusted) due to the non-normal distribution of the change scores, as confirmed by the Kolmogorov–Smirnov test, to investigate the hypothesized associations between GMV increases, symptom changes, and alexithymia throughout CBT. Additionally, two-sample *t*-tests (*p* < 0.05, one-tailed) were conducted to analyze the differences in GMV changes between patients who improved in their degree of remission of the depressive episode according to DSM-IV criteria and those who did not improve. We performed several robustness checks to detect potential effects of clinical variables of no interest on GMV and symptom changes, as well as analyses on associations between baseline levels and the change score of each variable (see Supplementary Information).

## Results

### Clinical effects of CBT

Depressive symptom burden decreased significantly within the patient group throughout CBT (BDI: *M*_Diff_ = 9.872, *t*(29) = 4.964, *p* < 0.001, *d* = 0.906, 95% CI [0.474, 1.327]; HDRS: *M*_Diff_ = 5.863, *t*(29) = 4.699, *p* < 0.001, *d* = 0.858, 95% CI [0.433, 1.272]), indicating its efficacy. DIF (*M*_Diff_ = 2.600, *t*(29) = 2.458, *p* = 0.020, *d* = 0.449, 95% CI [0.069, 0.821]) declined throughout CBT, while the TAS20 sum (*p* = 0.092), DDF (*p* = 0.695), and EOT (*p* = 0.323) did not change significantly. Nineteen of the MDD patients (63.3%) changed from acute to partially or fully remitted depressive episodes from t1 to t2 or from a partially remitted to a fully remitted depressive episode, respectively. TAS20 sum (*r*_s_ = 0.313, *p* = 0.046) and DIF (*r*_s_ = 0.339, *p* = 0.033) were positively correlated with BDI but uncorrelated with HDRS (*p* ≥ 0.144) at t1. There was no significant association between baseline DDF, EOT, and depressive symptoms (*p* ≥ 0.128). For detailed clinical results, see Supplementary Information (Supplementary Tables 1, 2).

### GMV changes

**Fig. 1:**
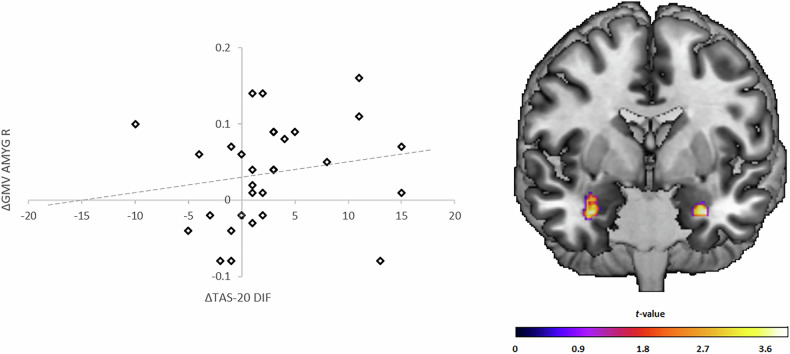
GMV changes in the amygdala within patients and the association with improvements in alexithymia (Difficulty Identifying Feelings). Left: Scatter plots depicting GMV changes (∆ = t2-t1) within the cluster of the right amygdala (x = 32, y = −3, z = −27) on the y-axis correlated with changes (∆ = t1-t2) in the Toronto Alexithymia Scale (TAS20) subscale Difficulty Identifying Feelings (DIF; *r*_s_ = 0.321, *p* = 0.042) on the x-axis within the patient group. Line: regression slope. Right: Coronal view (Montreal-Neurological-Institute coordinate y = 0) depicts the results of the paired *t*-tests (t1 vs. t2) in the patient group within the bilateral amygdala.

The mixed ANOVA analyses revealed a significant group x time interaction for the left amygdala (x = −27, y = 2, z = −26, *k* = 10, TFCE_(113)_ = 159.45_,_ *t*_(113)_ = 4.49, *p*_FWE_ = 0.041, η_p_^2^ = 0.198). Post Hoc analyses (hypothesis 1) revealed comprehensive GMV increases from t1 to t2 within patients. ROI analyses showed GMV increases in the bilateral amygdala (left: x = −28, y = 0, z = −27, *k* = 45, TFCE_(113)_ = 96.04_,_ *t*_(113)_ = 3.72, *p*_FWE_ = 0.020, *d* = 0.417; right: x = 32, y = −3, z = −27, *k* = 96, TFCE_(113)_ = 114.51_,_ *t*_(113)_ = 3.76, *p*_FWE_ = 0.015, *d* = 0.543, see Fig. 1) and the right anterior hippocampus (x = 30, y = −3, z = −27, *k* = 114, TFCE_(113)_ = 102.96_,_ *t*_(113)_ = 3.60, *p*_FWE_ = 0.022, *d* = 0.651). Additionally, significant GMV decreases from t1 to t2 were found in the right posterior hippocampus (x = 20, y = −22, z = −14, *k* = 474, TFCE_(113)_ = 141.04_,_ *t*_(113)_ = 3.24, *p*_FWE_ = 0.016, *d* = 0.751). The ROI analyses of the OFC (*p*_FWE_ ≥ 0.052) and the insula (*p*_FWE_ ≥ 0.079), as well as exploratory analyses on a whole brain level (*p*_FWE_ ≥ 0.053), revealed no significant effects. Cross-sectional analyses revealed no significant differences in GMV between patients and HC at t1 nor t2 for all ROIs and on a whole brain level (*p*_FWE_ ≥ 0.051).

### Associations between changes in GMV, symptoms, and alexithymia within patients

Regarding hypothesis 2, there was a small positive correlation between GMV increase in the cluster of the right amygdala and ∆DIF (*r*_s_ = 0.321, *p* = 0.042, see Fig. 1). However, concerning overall symptom improvement, for all extracted clusters, there were neither significant correlations of ∆GMV with ∆BDI (*p* ≥ 0.177) nor ∆HDRS (*p* ≥ 0.110). There were positive correlations of ΔDIF with ΔBDI (*r*_s_ = 0.388, *p* = 0.017) and ΔHDRS (*r*_s_ = 0.390, *p* = 0.017), as well as of ΔTAS20 sum with ΔBDI (*r*_s_ = 0.314, *p* = 0.046). All other associations between ∆GMV, ΔTAS20, and depressive symptom changes were not significant (*p* ≥ 0.061; see Supplementary Table 3 for details). There were no significant differences in GMV changes between patients with improvement in the degree of remission and those without (*p* ≥ 0.366). Besides, changes in these variables appeared robust against clinical variables of no interest (*p* ≥ 0.073; see Supplementary Information for details).

## Discussion

Using a naturalistic longitudinal neuroimaging design, the study demonstrated structural plasticity within limbic brain areas after CBT for MDD. As expected, GMV increased in parts of the bilateral amygdala and right hippocampus in patients. Unexpectedly, GMV decreases were observed in the right posterior hippocampus. As hypothesized, there was an association between limbic GMV increases and improvements in alexithymia, as those individuals who showed the highest volumetric increases within parts of the right amygdala tended to be the ones with the highest declines in DIF. However, an improvement in the severity of depressive symptoms in general was unrelated to any GMV changes.

Our results support the assumption that CBT affects not just the patient’s experiences and behavior but also brain structure and add to previous findings in depressive disorders [17, 18] and other mental disorders (e.g., social anxiety disorder: [40]; obsessive-compulsive disorder: [41]; spider phobia: [42]) that demonstrated the structural plastic effects of CBT. The current study underlines that therapy-induced changes in mental experiences may be significant or enduring enough to alter brain structure in line with Eric Kandel’s fifth principle [16]. In particular, the hippocampus-amygdala complex showed volume increases, the brain region where the most consistent brain structural effects were also observed in ECT [15]. Similar to a study on ECT-dependent GMV changes by Joshi et al. [43], hippocampal volume growth primarily occurred in the right anterior hippocampus. As highlighted by Joshi and colleagues, the anterior hippocampus appears to be crucially involved in affective disorders and their treatment due to its connections to areas pivotally involved in emotion processing and regulation. A recent study, moreover, revealed that a polygenetic risk for depression is significantly associated with smaller anterior but not posterior hippocampal volume in children and adolescents [44]. In line with the reported brain structural effects of ECT in MDD patients [15, 43], CBT was shown to be effective in increasing the GMV of the bilateral amygdala. Some studies, however, have reported amygdala enlargement [9–11] in acute depression. Therefore, some may argue that treatments should induce amygdala volume to decrease rather than increase. Nonetheless, as increases in amygdala volume were partly related to improvements in identifying feelings, they seem to express a shift to more adaptive emotion processing in the current MDD sample. Notably, patients showed only significant GMV changes within limbic but not cortico-limbic areas (here: insula, OFC). However, structural plasticity through antidepressants and ECT was also less often reported for cortico-limbic regions [15]. Compared to limbic areas, these areas are less prone to neuroplasticity, as even mature neurogenesis was found in the hippocampus and amygdala [45]. Moreover, limbic brain regions are directly involved in (emotional) learning and are, therefore, directly affected by molecular changes [46–48]. One unexpected result was the loss of GMV in parts of the right posterior hippocampus, contradicting a previously reported increase in posterior hippocampal volume in MDD patients due to AD [49]. Unlike the anterior parts, the posterior hippocampus is crucially involved in cognitive functions such as spatial memory [50]. Several studies have found diminished GMV in the posterior hippocampus in MDD [51–53], which was associated with cognitive impairments [49]. Similarly, in the healthy population, the posterior hippocampus is related to cognitive decline and is sensitive to age-related atrophy [54, 55]. As we did not assess neuropsychological symptoms, it remains unclear whether this result is due to a failure of CBT to improve cognitive functions or reflects an aging-related decline.

Our results show that CBT has structural effects on central brain regions for emotion processing (anterior hippocampus, amygdala). However, CBT’s capability to enhance emotion processing is often undermined as its core interventions primarily target externally observable behavior and cognitions. In fact, identifying emotions is an essential component of the most typical CBT methods, such as behavior analysis and cognitive restructuring. Baker et al. [56] demonstrated that CBT could diminish difficulties in emotion processing to a healthy degree and reduce overall alexithymia and its subdomains of DIF and DDF. The present findings match those of Baker [56], as they demonstrated not only volumetric increases in emotion-processing brain regions but also improvements in emotion identification on a behavioral level through CBT. Reductions in alexithymia and especially in DIF were positively correlated with treatment outcome, which was consistent with previous findings in sub-clinical depression [25]. The analyses also suggested a potential relationship between DIF and amygdala volume, which accords with reports from other studies [31]. However, those volumetric decreases were pronounced for the left amygdala [31], while they were only found for the right amygdala in the present study. While the left amygdala has been associated with more conscious emotion processing [57], the right amygdala is predominantly activated in automatic emotion processing, which, in turn, was also found to be impaired in alexithymia and DIF [58].

Opposite to previous studies on brain structural effects of CBT [17, 18], the present study did not find associations between GMV changes and improvement in overall depressive symptom severity. Thus, the current findings join the reported mixed findings in ECT, which sometimes found a correlation between brain structural effects (e.g., [43]) and overall clinical outcomes but several times failed to do so (e.g., [20, 59]). Since MDD is heterogeneous in nature, on a symptom, etiology, and, ultimately, on a neurobiological level [21, 22], consistent links between regional brain structure and global symptom effects tend to be challenging. Given the current results, it remains unclear whether GMV increases within the left amygdala and the right anterior hippocampus were only epiphenomena of CBT and, therefore, lack any association with symptom improvements or if we did not assess (dys-)functions specific to those regions in MDD.

Noteworthy strengths of the present study are the ecological validity, supported by the naturalistic design, the focus on (cortico-)limbic brain regions known for structural alterations in MDD, the relatively long interval or CBT density respectively, and the inclusion of alexithymia as a pivotal factor for MDD and psychotherapy, besides overall symptom severity. However, some limitations need to be considered. Firstly, as a depressed waitlist control group was not obtained, we could not account for the effects of the natural course of the disease. Secondly, due to the naturalistic design and the lack of information on which interventions have been carried out in CBT, we cannot connect GMV increase to specific interventions. Future research should target this problem by employing randomized control trials. Thirdly, even though the sample size of the present study is slightly larger than that of previous studies in this field, statistical power is still limited to detect potentially smaller effects. Fourthly, as correlations between GMV increases and enhancements in specific psychological functions (right amygdala GMV and emotion identification) were only small, they need to be interpreted with caution. On a trend level, they provide helpful insight into the potential underlying mechanism of how CBT could impact GMV in patients with MDD. However, a significant portion of the variance in GMV changes remains unexplained. We encourage future research to explore additional symptoms and dysfunctions related to MDD, such as sleep patterns, executive functions, rumination, or anhedonia, and their potential connections to the structural brain changes induced by psychotherapy. Fifthly, even though our time interval was longer than in previous work, it remains unclear how sustainable the structural effects are beyond the duration of CBT. Therefore, studies on the long-term brain structural effects of CBT or psychotherapy in general on MDD should be conducted in the future, following the example of existing studies concerning other mental disorders [60] or other treatments for depressive disorders (ECT: [20]).

In sum, the present study demonstrated the capability of CBT to have associated effects on regional limbic GMV in MDD and, therefore, delivered a measurable biomarker of psychotherapy that could add to its efficacy. In line with previous work, the findings show that brain structure in MDD is potentially modifiable, not just through somatic treatments but also cognitive-behavioral changes, which could benefit patients’ self-efficacy. The revealed missing link of GMV changes throughout CBT and improvements in overall depressive symptom severity and the simultaneous finding of connection with improvements in specific (dys)functions emphasize the need for clinical-neuroscientific research focusing more on how specific brain areas relate to maladaptive behavior. Understanding the psychopathological and neurobiological heterogeneity within MDD might be promising in terms of personalized healthcare, intending to optimize clinical outcomes.

## Supplementary information

Supplementary Information

## Data Availability

The data that support the findings of this study are available from the corresponding author upon reasonable request.
