# Supplementary material for: Limbic gray matter increases in response to cognitive-behavioral therapy in major depressive disorder
Source: Transl Psychiatry. 2025 Aug 27;15:301. doi: 10.1038/s41398-025-03545-7 (PMC12381077; doi:10.1038/s41398-025-03545-7)
Supplement: Supplementary file 1 — Supplementary Information [file 41398_2025_3545_MOESM1_ESM.docx]

**Supplementary Information**

**Clinical effects of CBT – detailed results**

**Supplementary Table 1**

| clinical variable |  | *t1* | | *t2* | |  |  |  |  |
| --- | --- | --- | --- | --- | --- | --- | --- | --- | --- |
|  |  | *M* | *SD* | *M* | *SD* | *t*(29) | *p* | *d* | 95% CI |
| BDI |  | 23.37 | 10.27 | 13.50 | 8.73 | 4.96 | <.001 | 0.906 | [0.474, 1.327] |
| HDRS |  | 13.53 | 5.73 | 7.67 | 5.17 | 4.70 | <.001 | 0.858 | [0.433, 1.272] |
| TAS20 |  |  |  |  |  |  |  |  |  |
| sum |  | 54.45 | 11.12 | 50.88 | 10.86 | 1.74 | .092 | 0.318 | [-0.052, 0.682] |
| DIF |  | 20.27 | 6.33 | 17.67 | 5.30 | 2.46 | .020 | 0.449 | [0.069, 0.821] |
| DDF |  | 15.43 | 4.18 | 15.10 | 4.44 | 0.40 | .695 | 0.072 | [-0.287, 0.430] |
| EOT |  | 18.77 | 4.06 | 18.13 | 4.54 | 1.01 | .323 | 0.184 | [-0.179, 0.543] |

*Results of the paired t-tests of changes in clinical variables from t1 to t2 within patients*

*Note*. *n*=30 patients. BDI=Beck’s Depression Inventory. HDSR=Hamilton-Depression-Rating-Scale. TAS20=Toronto Alexithymia Scale. DIF=Difficulty Identifying Feelings. DDF=Difficulty Describing Feelings. EOT=Externally-Oriented Thinking. *d*=Cohen’s *d*. All *p* values for the post hoc two-sample *t*-test are two-tailed.

**Supplementary Table 2**

| clinical variable | 1 | 2 | 3 | 4 | 5 | 6 |
| --- | --- | --- | --- | --- | --- | --- |
| 1. BDI | - | - | - | - | - | - |
| 2. HDRS | **.693** | - | - | - | - | - |
| 3. TAS20 sum | **.313** | .201 | - | - | - | - |
| 4. DIF | **.339** | .190 | **.869** | - | - | - |
| 5. DDF | .177 | .011 | **.677** | **.529** | - | - |
| 6. EOT | .126 | .214 | .**589** | .243 | .132 | - |

*Spearman correlations for clinical variables within patients at t1*

*Note*. *n*=30 patients. BDI=Beck’s Depression Inventory. HDSR=Hamilton-Depression-Rating-Scale. TAS20=Toronto Alexithymia Scale. DIF=Difficulty Identifying Feelings. DDF=Difficulty Describing Feelings. EOT=Externally-Oriented Thinking. Bold correlation coefficients indicate *p*<.05 (one-tailed).

**Associations between changes in GMV, symptoms, and alexithymia within patients – detailed results**

**Supplementary Table 3**

| variable | 1 | 2 | 3 | 4 | 5 | 6 | 7 | 8 | 9 | 10 |
| --- | --- | --- | --- | --- | --- | --- | --- | --- | --- | --- |
| 1. ΔBDI | - | - | - | - | - | - | - | - | - | - |
| 2. ΔHDRS | **.353** | - | - | - | - | - | - | - | - | - |
| 3. ΔTAS20 sum | **.314** | .289 | - | - | - | - | - | - | - | - |
| 4. ΔDIF | **.388** | **.390** | **.712** | - | - | - | - | - | - | - |
| 5. ΔDDF | .100 | .103 | **.862** | **.447** | - | - | - | - | - | - |
| 6. ΔEOT | .046 | -.048 | .**607** | .234 | **.607** | - | - | - | - | - |
| ΔGMV |  |  |  |  |  |  |  |  |  |  |
| 7. L Amyg | .121 | .270 | .207 | .196 | .103 | -.040 | - | - | - | - |
| 8. R Amyg | .060 | .153 | .282 | **.321** | .135 | -.160 | .153 | - | - | - |
| 9. R Hippocampus (anter.) | -.267 | -.001 | .148 | .150 | .104 | -.168 | .066 | **.691** | - | - |
| 10. R Hippocampus (post.) | -.152 | .082 | -.122 | -.073 | -.076 | .059 | -.079 | -.079 | -.096 | - |

*Spearman correlations for changes in GMV, symptoms and alexithymia from t1 to t2 within patients*

*Note*. *n*=30 patients. BDI=Beck’s Depression Inventory. HDSR=Hamilton-Depression-Rating-Scale. TAS20=Toronto Alexithymia Scale. DIF=Difficulty Identifying Feelings. DDF=Difficulty Describing Feelings. EOT=Externally-Oriented Thinking. GMV=gray matter volume. L=left brain hemisphere. R=right brain hemisphere. Amyg=Amygdala. anter.=anterior. post.=posterior. Bold correlation coefficients indicate *p*<.05 (one-tailed).

**Supplementary Table 4**

| variable | Δ1 | Δ2 | Δ3 | Δ4 | Δ5 | Δ6 | Δ7 | Δ8 | Δ9 | Δ10 |
| --- | --- | --- | --- | --- | --- | --- | --- | --- | --- | --- |
| 1. BDI | .**469** | .232 | .069 | .158 | -.066 | -.257 | -.145 | .101 | -.068 | -.078 |
| 2. HDRS | **.312** | **.632** | .090 | .289 | -.097 | -.243 | .019 | .204 | .042 | .119 |
| 3. TAS20 sum | **.321** | .245 | **.518** | **.451** | **.388** | .241 | .217 | -.061 | -.115 | -.066 |
| 4. DIF | .292 | .108 | **.480** | **.508** | .273 | .196 | .041 | .009 | -.002 | -.058 |
| 5. DDF | .024 | .024 | **.394** | .110 | **.546** | .206 | .089 | -.114 | .032 | -.050 |
| 6. EOT | **.328** | **.326** | .254 | .270 | .254 | .252 | .295 | -.184 | **-.423** | -.076 |
| GMV |  |  |  |  |  |  |  |  |  |  |
| 7. L Amyg | -.176 | **-.337** | -.159 | -.170 | -.119 | .069 | **-.924** | .015 | .083 | -.066 |
| 8. R Amyg | -.059 | **-.363** | -.258 | **-.368** | -.096 | .231 | -.251 | **-.566** | -.303 | -.203 |
| 9. R Hippocampus (anter.) | -.133 | -.281 | -.221 | -.273 | -.149 | .126 | -.217 | -.126 | -.248 | -.256 |
| 10. R Hippocampus (post.) | -.014 | **-.421** | .093 | .015 | .120 | .113 | -.168 | .130 | .086 | **-.570** |

*Spearman correlations for baseline levels (t1) and changes from t1 to t2 in GMV, symptoms and alexithymia within patients*

*Note*. *n*=30 patients. Δ=change from t1 to t2. BDI=Beck’s Depression Inventory. HDSR=Hamilton-Depression-Rating-Scale. TAS20=Toronto Alexithymia Scale. DIF=Difficulty Identifying Feelings. DDF=Difficulty Describing Feelings. EOT=Externally-Oriented Thinking. GMV=gray matter volume. L=left brain hemisphere. R=right brain hemisphere. Amyg=Amygdala. anter.=anterior. post.=posterior. Bold correlation coefficients indicate *p*<.05 (one-tailed).

**Robustness checks**

*Methods*: To detect the effects of clinical variables of no interest on GMV and symptom changes, we performed Spearman correlations (*p*<.05, two-tailed) for medication load index at t1, medication load index change (Δ=t2-t1), and number of CBT sessions. Potential effects of medication status (medicated vs. non-medicated), comorbidity, degree of remission, and recurrence of MDD episodes at t1 were tested by applying two-sample t-tests (*p*<.05, two-tailed).

*Results*: ∆GMV, ∆BDI, ∆HDRS, or ∆TAS20 were not significantly associated with the medication load index at t1 (*p*≥.104), ∆medication load index from t1 to t2 (*p*≥.198), and the numbers of CBT sessions at t2 (*p*≥.272). Medication status (*p*≥.068), comorbidity (*p*≥.169), degree of remission (*p*≥.077), and recurrence of MDD episodes (*p*≥.073) at t1 did not have significant effects on ∆GMV, ∆BDI, ∆HDRS, or ∆TAS20.
